# The genome sequence of the White-barred Gold, *Micropterix aruncella* (Scopoli, 1763)

**DOI:** 10.12688/wellcomeopenres.18714.1

**Published:** 2023-01-05

**Authors:** Peter W. H. Holland, James Hammond, Amanda S. Holland, University of Oxford and Wytham Woods Genome Acquisition Lab, Darwin Tree of Life Barcoding collective, Wellcome Sanger Institute Tree of Life programme, Wellcome Sanger Institute Scientific Operations: DNA Pipelines collective, Tree of Life Core Informatics collective, Darwin Tree of Life Consortium

**Affiliations:** 1 University of Oxford, Oxford, Oxfordshire, UK

**Keywords:** Micropterix aruncella, White-barred Gold, genome sequence, chromosomal, Lepidoptera

## Abstract

We present a genome assembly from an individual female *Micropterix aruncella* (the White-barred Gold; Arthropoda, Insecta, Lepidoptera; Micropterigidae). The genome sequence is 1,079 megabases in span. Most of the assembly is scaffolded into 31 chromosomal pseudomolecules, including the assembled Z sex chromosome. The mitochondrial genome has also been assembled and is 15.0 kilobases in length.

## Species taxonomy

Eukaryota; Metazoa; Ecdysozoa; Arthropoda; Hexapoda; Insecta; Pterygota; Neoptera; Endopterygota; Lepidoptera; Zeugloptera; Micropterigidae; *Micropterix*; *Micropterix aruncella* (Scopoli, 1763) (NCBI:txid1042620).

## Background

The phylogenetic relationships between families of Lepidoptera have been the subject of debate and discussion for decades, with much uncertainty. One point of almost total agreement, from the earliest morphological analyses to the latest molecular trees, is that the family Micropterygidae is the extant sister group to all other Lepidoptera (with the rare family Agathiphagidae sometimes placed sister to Micropterygidae ( Regier *et al.*, 2013; Regier *et al.*, 2015). Therefore, the most ancient node in lepidopteran phylogeny separates Micropterygidae from all other moths and butterflies. In order to catalogue and understand the molecular innovations that characterise all Lepidoptera, it is essential to include genomic data from moths in this family. Li *et al.* (2021) previously reported the genome of *Neomicropteryx cornuta*, but there is a need for additional genome sequence data from the Micropterygidae.

*Micropterix aruncella* (White-barred Gold) is a small day-flying moth in the family Micropterygidae, with a scattered distribution across the UK, Europe and Russia ( *National Biodiversity Atlas*, no date; GBIF Secretariat, 2021). Adults have a wingspan of only 6–7 mm. Males are recognisable by their bright golden colouration crossed by two silver stripes, while females lack the stripes and can be difficult to distinguish from some other *Micropterix* species. Sex determination in the genus *Micropterix* is reported to involve a Z/ZZ chromosome system ( Traut & Marec, 1997). Like other members of the family, adult *M. aruncella* lack a proboscis: adults feed on pollen using chewing mouthparts, often from buttercup flowers in sunny woodland glades. Larvae are thought to feed at the base of herbaceous plants, but much remains to be learnt about their habits. The anatomy of the larva has been described in detail by ( Klausnitzer *et al.*, 2002).

The genome of *M. aruncella* was sequenced as part of the Darwin Tree of Life Project, a collaborative effort to sequence all named eukaryotic species in the Atlantic Archipelago of Britain and Ireland. Here we present a chromosomally complete genome sequence for *M. aruncella*, based on the ilMicArun2 specimen from Bagley Wood, Oxfordshire, UK.

## Genome sequence report

**Figure 1.**
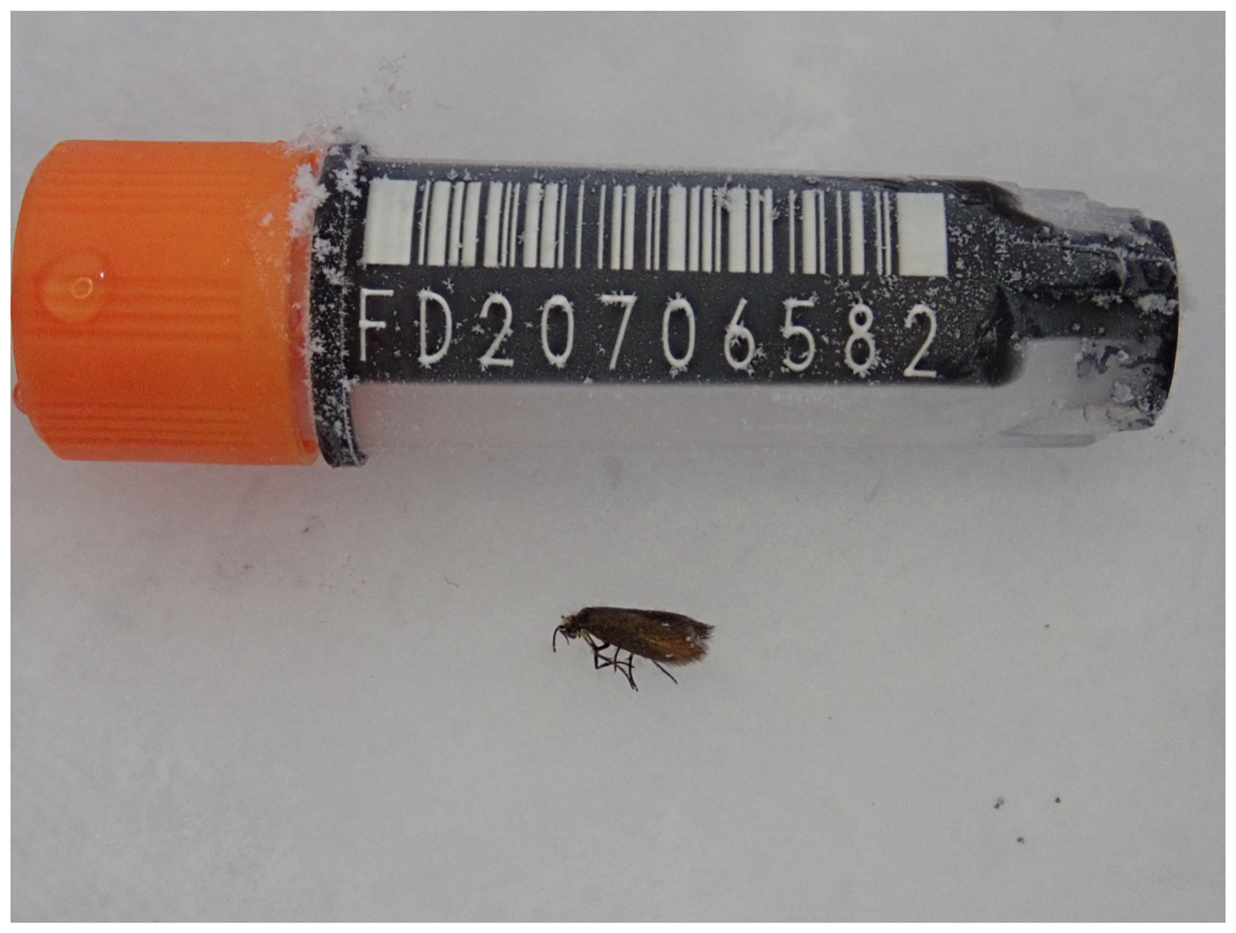
Photograph of the *Micropterix aruncella* (ilMicArun2) specimen used for genome sequencing.

The genome was sequenced from a female *M. aruncella* specimen ( Figure 1) collected from Bagley Wood, Berkshire, UK (51.72, –1.27). A total of 18-fold coverage in Pacific Biosciences single-molecule HiFi long reads was generated. Primary assembly contigs were scaffolded with chromosome conformation Hi-C data. Manual assembly curation corrected 462 missing or mis-joins and removed 75 haplotypic duplications, reducing the assembly length by 0.8% and the scaffold number by 15.8%, and increasing the scaffold N50 by 5.29%.

**Figure 2.**
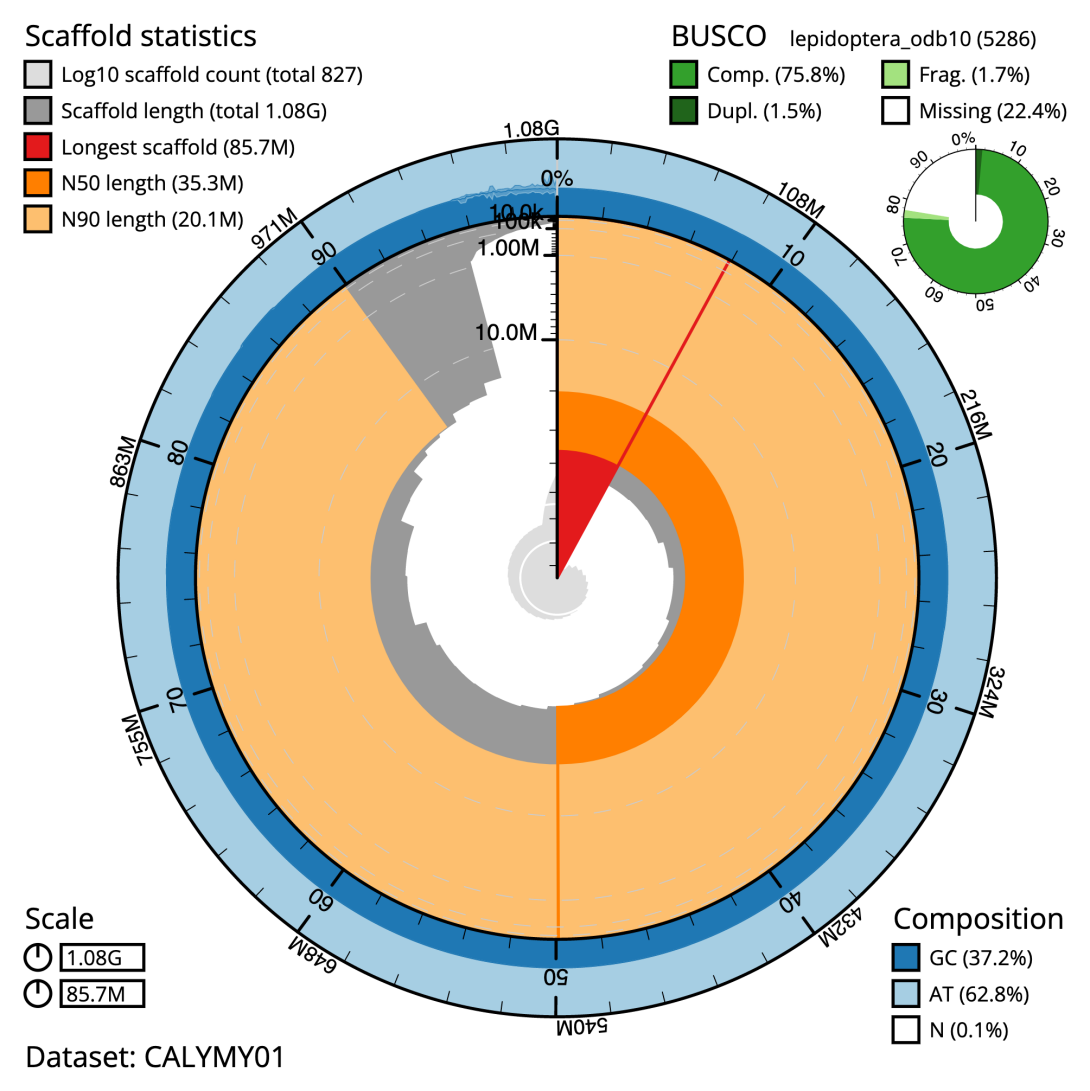
Genome assembly of *Micropterix aruncella*, ilMicArun2.1: metrics. The BlobToolKit Snailplot shows N50 metrics and BUSCO gene completeness. The main plot is divided into 1,000 size-ordered bins around the circumference with each bin representing 0.1% of the 1,079,211,800 bp assembly. The distribution of scaffold lengths is shown in dark grey with the plot radius scaled to the longest scaffold present in the assembly (85,726,809 bp, shown in red). Orange and pale-orange arcs show the N50 and N90 scaffold lengths (35,295,981 and 20,073,628 bp), respectively. The pale grey spiral shows the cumulative scaffold count on a log scale with white scale lines showing successive orders of magnitude. The blue and pale-blue area around the outside of the plot shows the distribution of GC, AT and N percentages in the same bins as the inner plot. A summary of complete, fragmented, duplicated and missing BUSCO genes in the lepidoptera_odb10 set is shown in the top right. An interactive version of this figure is available at https://blobtoolkit.genomehubs.org/view/ilMicArun2.1/dataset/CALYMY01/snail.

The final assembly has a total length of 1,079.2 Mb in 826 sequence scaffolds with a scaffold N50 of 35.3 Mb ( Table 1). Most (95.96%) of the assembly sequence was assigned to 31 chromosomal-level scaffolds, representing 30 autosomes and the Z sex chromosome. Chromosome-scale scaffolds confirmed by the Hi-C data are named in order of size ( Figure 2– Figure 5; Table 2). While not fully phased, the assembly deposited is of one haplotype. Contigs corresponding to the second haplotype have also been deposited.

**Table 1.** Genome data for *Micropterix aruncella*, ilMicArun2.1.

Project accession data		
Assembly identifier	ilMicArun2.1	
Species	*Micropterix aruncella*	
Specimen	ilMicArun2; ilMicArun4	
NCBI taxonomy ID	1042620	
BioProject	PRJEB53243	
BioSample ID	SAMEA10978742	
Isolate information	female (PacBio);	
Assembly metrics *		*Benchmark*
Consensus quality (QV)	53.9	*≥ 50*
*k*-mer completeness	99.98%	*≥ 95%*
BUSCO **	C:75.8%[S:74.4%,D:1.5%], F:1.7%,M:22.4%,n:5286	*C ≥ 95%*
Percentage of assembly mapped to chromosomes	95.96%	*≥ 95%*
Sex chromosomes	Z	*localised homologous* *pairs*
Organelles	Mitochondrial genome assembled	*complete single alleles*
Raw data accessions		
PacificBiosciences SEQUEL II	ERR9836424	
Hi-C Illumina	ERR9820269	
Genome assembly		
Assembly accession	GCA_945859715.1	
*Accession of alternate haplotype*	GCA_945859755.1	
Span (Mb)	1079.2	
Number of contigs	4,032	
Contig N50 length (Mb)	0.5	
Number of scaffolds	826	
Scaffold N50 length (Mb)	35.3	
Longest scaffold (Mb)	85.7	

* Assembly metric benchmarks are adapted from column VGP-2020 of “Table 1: Proposed standards and metrics for defining genome assembly quality” from ( Rhie *et al.*, 2021). ** BUSCO scores based on the lepidoptera_odb10 BUSCO set using v5.3.2. C = complete [S = single copy, D = duplicated], F = fragmented, M = missing, n = number of orthologues in comparison. A full set of BUSCO scores is available at https://blobtoolkit.genomehubs.org/view/ilMicArun2.1/dataset/CALYMY01/busco.

**Figure 3.**
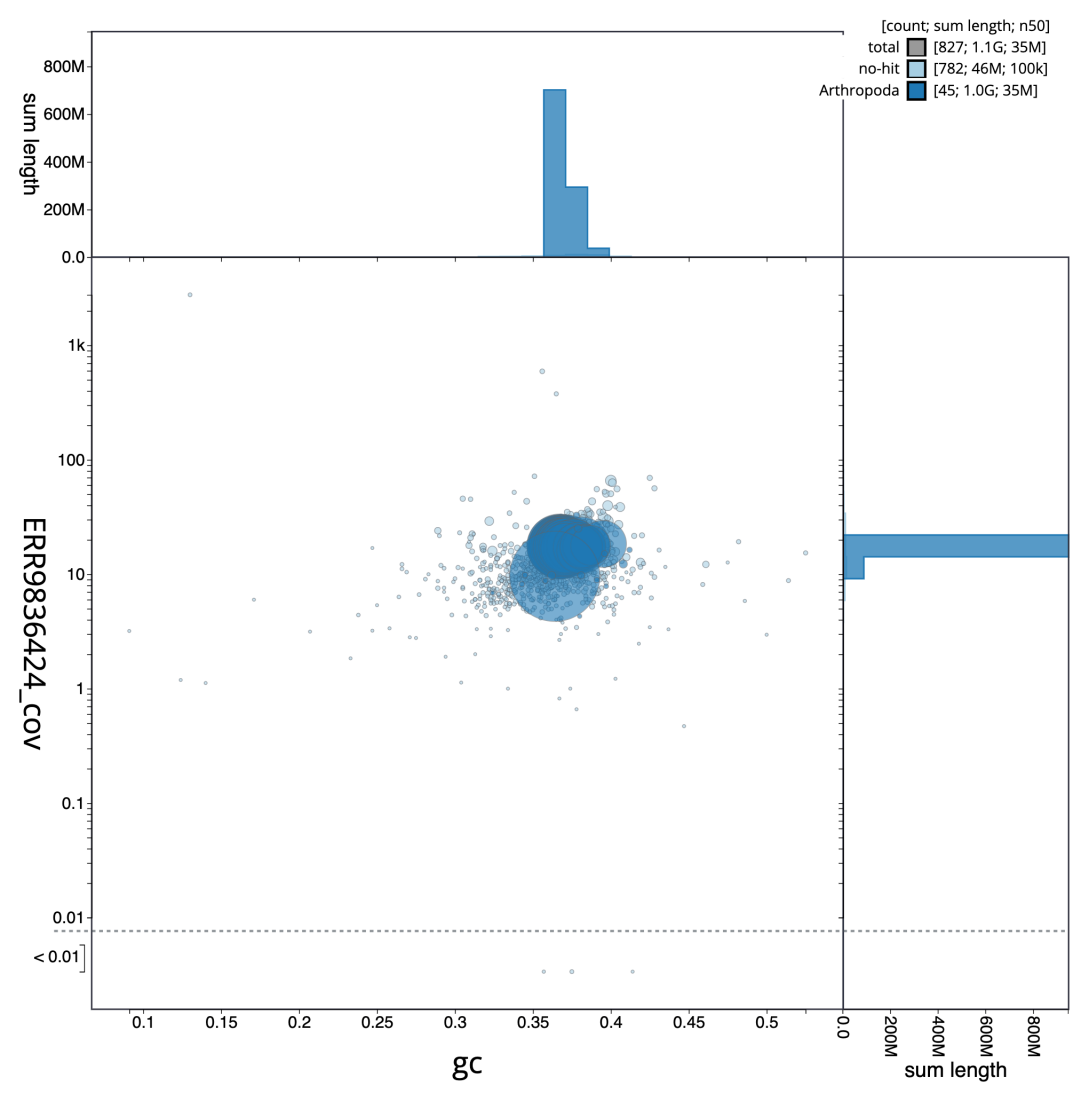
Genome assembly of *Micropterix aruncella*, ilMicArun2.1: GC coverage. BlobToolKit GC-coverage plot. Scaffolds are coloured by phylum. Circles are sized in proportion to scaffold length. Histograms show the distribution of scaffold length sum along each axis. An interactive version of this figure is available at https://blobtoolkit.genomehubs.org/view/ilMicArun2.1/dataset/CALYMY01/blob.

**Figure 4.**
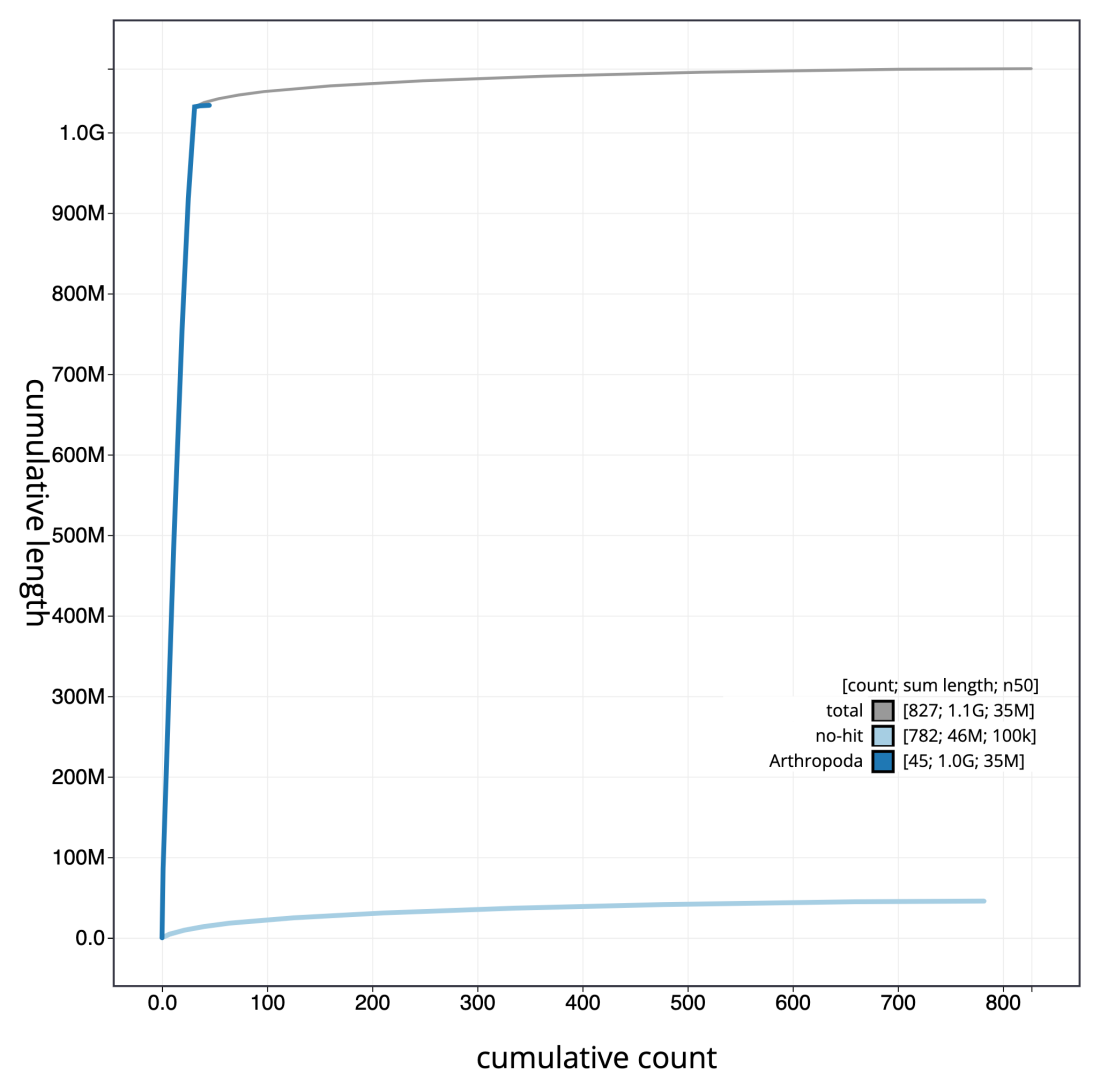
Genome assembly of *Micropterix aruncella*, ilMicArun2.1: cumulative sequence. BlobToolKit cumulative sequence plot. The grey line shows cumulative length for all scaffolds. Coloured lines show cumulative lengths of scaffolds assigned to each phylum using the buscogenes taxrule. An interactive version of this figure is available at https://blobtoolkit.genomehubs.org/view/ilMicArun2.1/dataset/CALYMY01/cumulative.

**Figure 5.**
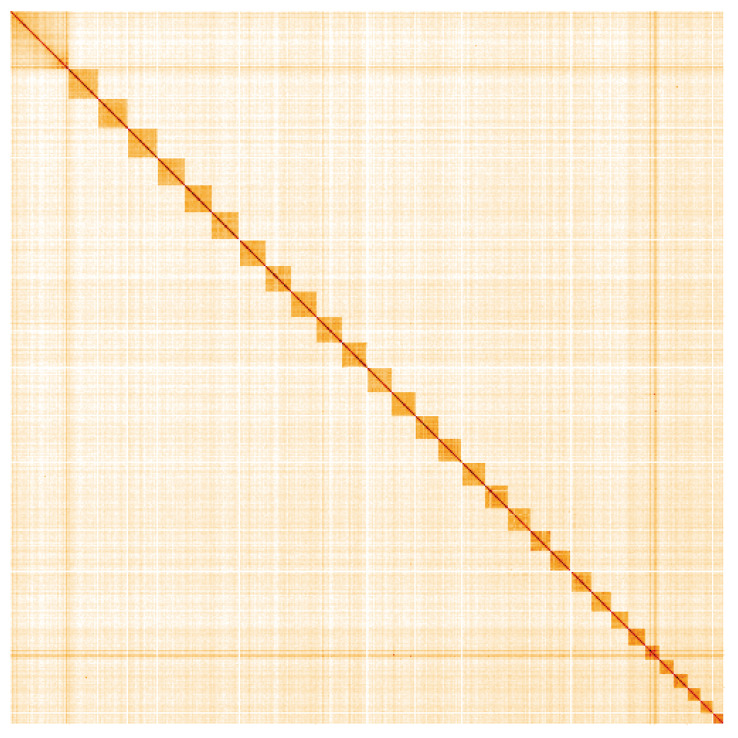
Genome assembly of *Micropterix aruncella*, ilMicArun2.1: Hi-C contact map. Hi-C contact map of the ilMicArun2.1 assembly, visualised using HiGlass. Chromosomes are shown in order of size from left to right and top to bottom. An interactive version of this figure may be viewed at https://genome-note-higlass.tol.sanger.ac.uk/l/?d=OyCMTRpkQFm2ratZEtMKpA.

**Table 2.** Chromosomal pseudomolecules in the genome assembly of *Micropterix aruncella*, ilMicArun2.

INSDC accession	Chromosome	Size (Mb)	GC%
OX155950.1	1	42.65	36.7
OX155951.1	2	42.44	36.8
OX155952.1	3	42.26	36.8
OX155953.1	4	40.56	36.7
OX155954.1	5	39.16	37.2
OX155955.1	6	39.14	36.6
OX155956.1	7	37.96	36.7
OX155957.1	8	37.37	36.9
OX155958.1	9	36.79	36.9
OX155959.1	10	36.75	36.8
OX155960.1	11	35.92	37
OX155961.1	12	35.3	36.9
OX155962.1	13	34.31	36.9
OX155963.1	14	33.76	37
OX155964.1	15	33.7	37
OX155965.1	16	33.51	37.1
OX155966.1	17	33.39	37.2
OX155967.1	18	32.43	38.1
OX155968.1	19	29.16	37.7
OX155969.1	20	29.1	37.4
OX155970.1	21	28.6	37.6
OX155971.1	22	28.55	37.1
OX155972.1	23	24.8	37.1
OX155973.1	24	24.62	37.9
OX155974.1	25	21.75	39.5
OX155975.1	26	20.69	38
OX155976.1	27	20.07	38.2
OX155977.1	28	18.17	38.3
OX155978.1	29	17.82	38.1
OX155979.1	30	15.34	38.7
OX155980.1	Z	85.73	36.4
OX155981.1	MT	0.01	13
-	-	47.4	37.2

The assembly has a BUSCO v5.3.2 ( Manni *et al.*, 2021) completeness of 75.8% (single 74.4%, duplicated 1.5%), using the OrthoDB version 10 lepidoptera reference set This test may not be optimal for *M. aruncella* as the Micropterygidae are an outgroup to the set of species used to generate the Lepidoptera reference set. Using the Insecta reference set, the BUSCO completeness is 97.7% (single 96.0%, duplicated 1.7%). Evaluation of the assembly shows a consensus quality value (QV) of 53.9 and *k*-mer completeness of 99.98%.

## Methods

### Sample acquisition and nucleic acid extraction

A series of male and female *M. aruncella* (ilMicArun2) specimens were collected in Bagley Wood, Oxfordshire (Biological vice-county: Berkshire), UK (latitude 51.72, longitude –1.27) by Peter Holland, James Hammond and Amanda Holland (all University of Oxford) by daytime searching and netting. Specimens were identified by James Hammond and snap-frozen at –80°C by Peter Holland. Specimen ilMicArun2 (female) was used for acquisition of the genome sequence; specimen ilMicArun4 (male) was used for Hi-C scaffolding.

DNA was extracted at the Tree of Life laboratory, Wellcome Sanger Institute. The ilMicArun2 sample was weighed and dissected on dry ice with tissue set aside for Hi-C sequencing. Whole body tissue was cryogenically disrupted to a fine powder using a Covaris cryoPREP Automated Dry Pulveriser, receiving multiple impacts. High molecular weight (HMW) DNA was extracted using the Qiagen MagAttract HMW DNA extraction kit. HMW DNA was sheared into an average fragment size of 12–20 kb in a Megaruptor 3 system with speed setting 30. Sheared DNA was purified by solid-phase reversible immobilisation using AMPure PB beads with a 1.8X ratio of beads to sample to remove the shorter fragments and concentrate the DNA sample. The concentration of the sheared and purified DNA was assessed using a Nanodrop spectrophotometer and Qubit Fluorometer and Qubit dsDNA High Sensitivity Assay kit. Fragment size distribution was evaluated by running the sample on the FemtoPulse system.

### Sequencing

Pacific Biosciences HiFi circular consensus and 10X Genomics read cloud DNA sequencing libraries were constructed according to the manufacturers’ instructions. DNA sequencing was performed by the Scientific Operations core at the WSI on the Pacific Biosciences SEQUEL II (HiFi) instruments. Hi-C data were also generated from whole body tissue of ilMicArun4 using the Arima v2 kit and sequenced on the Illumina NovaSeq 6000 instrument.

### Genome assembly

Assembly was carried out with Hifiasm ( Cheng *et al.*, 2021) and haplotypic duplication was identified and removed with purge_dups ( Guan *et al.*, 2020). The assembly was scaffolded with Hi-C data ( Rao *et al.*, 2014) using YaHS ( Zhou *et al.*, 2022). The assembly was checked for contamination and corrected using the gEVAL system ( Chow *et al.*, 2016) as described previously ( Howe *et al.*, 2021). Manual curation was performed using gEVAL, HiGlass ( Kerpedjiev *et al.*, 2018) and Pretext ( Harry, 2022). The mitochondrial genome was assembled using MitoHiFi ( Uliano-Silva *et al.*, 2021), which performed annotation using MitoFinder ( Allio *et al.*, 2020). The genome was analysed and BUSCO scores generated within the BlobToolKit environment ( Challis *et al.*, 2020). Table 3 contains a list of all software tool versions used, where appropriate.

**Table 3.** Software tools and versions used.

Software tool	Version	Source
BlobToolKit	3.4.0	Challis *et al.*, 2020
gEVAL	N/A	Chow *et al.*, 2016
Hifiasm	0.16.1-r375	Cheng *et al.*, 2021
HiGlass	1.11.6	Kerpedjiev *et al.*, 2018
MitoHiFi	2	Uliano-Silva *et al.*, 2021
PretextView	0.2	Harry, 2022
purge_dups	1.2.3	Guan *et al.*, 2020
YaHS	yahs-1.1.91eebc2	Zhou *et al.*, 2022

### Ethics/compliance issues

The materials that have contributed to this genome note have been supplied by a Darwin Tree of Life Partner. The submission of materials by a Darwin Tree of Life Partner is subject to the Darwin Tree of Life Project Sampling Code of Practice. By agreeing with and signing up to the Sampling Code of Practice, the Darwin Tree of Life Partner agrees they will meet the legal and ethical requirements and standards set out within this document in respect of all samples acquired for, and supplied to, the Darwin Tree of Life Project. Each transfer of samples is further undertaken according to a Research Collaboration Agreement or Material Transfer Agreement entered into by the Darwin Tree of Life Partner, Genome Research Limited (operating as the Wellcome Sanger Institute), and in some circumstances other Darwin Tree of Life collaborators.

## Data Availability

European Nucleotide Archive: *Micropterix aruncella* (white-barred gold). Accession number PRJEB53243; https://identifiers.org/ena.embl/PRJEB53243 ( Wellcome Sanger Institute, 2022) The genome sequence is released openly for reuse. The *Micropterix aruncella* genome sequencing initiative is part of the Darwin Tree of Life (DToL) project. All raw sequence data and the assembly have been deposited in INSDC databases. The genome will be annotated and presented through the Ensembl pipeline at the European Bioinformatics Institute. Raw data and assembly accession identifiers are reported in Table 1.

## Author information

Members of the University of Oxford and Wytham Woods Genome Acquisition Lab are listed here: https://doi.org/10.5281/zenodo.4789928.

Members of the Darwin Tree of Life Barcoding collective are listed here: https://doi.org/10.5281/zenodo.4893703.

Members of the Wellcome Sanger Institute Tree of Life programme are listed here: https://doi.org/10.5281/zenodo.4783585.

Members of Wellcome Sanger Institute Scientific Operations: DNA Pipelines collective are listed here: https://doi.org/10.5281/zenodo.4790455.

Members of the Tree of Life Core Informatics collective are listed here: https://doi.org/10.5281/zenodo.5013541.

Members of the Darwin Tree of Life Consortium are listed here: https://doi.org/10.5281/zenodo.4783558.
