# Supplementary material for: The genome sequence of the White-barred Gold, Micropterix aruncella (Scopoli, 1763)
Source: Wellcome Open Res. 2023 Jan 5;8:1. [Version 1] doi: 10.12688/wellcomeopenres.18714.1 (PMC10288161; doi:10.12688/wellcomeopenres.18714.1)
Supplement: Peer review history

# Peer review: Reviewer response for version 1

**DOI:** 10.21956/wellcomeopenres.20752.r58576

**Published:** 2023-06-22

**Authors:** Rodolpho S. T. Menezes

**Affiliations:** 1 Department of Biological Sciences, State University of Santa Cruz, Ilhéus, Brazil

This genome report describes the genome sequencing of the white-barred gold, *Micropterix aruncella*. This genome will be interesting for comparative genomics investigation within Lepidoptera. Moreover, the manuscript is well written and therefore it can be indexed. Below I list a few comments to the authors to improve the manuscript:

- “The phylogenetic relationships between…”; “The phylogenetic relationships among…”.
- “detail by (Klausnitzer et al., 2002)”; “detail by Klausnitzer et al., 2002”.
- Genome assembly: Since you assembled mitochondrial genome I think it is interesting you show general (GC/AT content…) mitochondrial information about this species.
- Table 3: Please include MitoFinder information.

Are sufficient details of methods and materials provided to allow replication by others?

Yes

Is the rationale for creating the dataset(s) clearly described?

Yes

Are the datasets clearly presented in a useable and accessible format?

Yes

Are the protocols appropriate and is the work technically sound?

Yes

Reviewer Expertise:

Cytogenetics, Phylogenomics and Phylogeography.

I confirm that I have read this submission and believe that I have an appropriate level of expertise to confirm that it is of an acceptable scientific standard.

# Peer review: Reviewer response for version 1

**DOI:** 10.21956/wellcomeopenres.20752.r58577

**Published:** 2023-05-30

**Authors:** Joel Vizueta

**Affiliations:** 1 University of Copenhaguen, Copenhaguen, Denmark

The authors describe the genome assembly of *Micropterix aruncella* in the submitted data note, which is a high quality phased genome assembled at the chromosome level. This genome is a valuable resource for phylogenomic and comparative genomics analyses as an outgroup of Lepidoptera. 

 The methods are robust, and I would recommend this manuscript to be accepted although I have some suggestions that could improve the manuscript:

- Table 1: The BUSCO score calls the attention as it is very low for a high quality genome, so I would recommend to add another row with the Insecta BUSCO dataset so the reader can see that it might just be an artifact of the Lepidopera dataset. The authors discuss it in the text, but I would add it in the table. In addition, they could add a more general BUSCO dataset, such as Eukaryota to validate the gene completeness.
- I see in the dTol webpage that this genome is already annotated and the annotations are publicly available. The authors need to update this information in the text, and I would encourage to include the statistics from gene annotation in the manuscript. 

Are sufficient details of methods and materials provided to allow replication by others?

Yes

Is the rationale for creating the dataset(s) clearly described?

Yes

Are the datasets clearly presented in a useable and accessible format?

Yes

Are the protocols appropriate and is the work technically sound?

Yes

Reviewer Expertise:

Genomics

I confirm that I have read this submission and believe that I have an appropriate level of expertise to confirm that it is of an acceptable scientific standard.
